# Impact of Chronic Kidney Disease on the Coronary Revascularization Guided by Intracoronary Physiology: Results of the First Registry with Long-Term Follow-Up in a Latin American Population

**DOI:** 10.3390/jcdd11070216

**Published:** 2024-07-10

**Authors:** Clarissa Campo Dall’Orto, Rubens Pierry Ferreira Lopes, Lara Vilela Eurípedes, Gilvan Vilella Pinto Filho, Marcos Raphael da Silva

**Affiliations:** Department of Hemodynamic and Interventional Cardiology, Advanced Hemodynamic Therapy Center, Brazilian Society of Health Support Hospital, Teixeira de Freitas 45987-088, BA, Brazil

**Keywords:** coronary disease, coronary stent, fractional flow reserve

## Abstract

The use of invasive physiology methods in patients with renal dysfunction is not well elucidated. Our objective was to evaluate the in-hospital and long-term results of using intracoronary physiology to guide revascularization in patients with chronic kidney disease. In this retrospective study, we evaluated 151 patients from January 2018 to January 2022, divided into 2 groups: CKD (81 patients [114 lesions]) and non-CKD (70 patients [117 lesions]). The mean age was higher (*p* < 0.001), body mass index was lower (*p* = 0.007), contrast volume used was lower (*p* = 0.02) and the number of ischemic lesions/patients was higher (*p* = 0.005) in the CKD group. The primary outcomes (rate of major adverse cardiac events during follow-up, defined as death, infarction, and need for new revascularization) in the CKD and non-CKD groups were 22.07% and 14.92%, respectively (*p* = 0.363). There was a significant difference in the target lesion revascularization (TLR) rate (11.68%, CKD group vs. 1.49%, non-CKD group, *p* = 0.02), this initial statistical difference was not significant after adjusting for variables in the logistic regression model. There was no difference between the rates of death from all causes (6.49%, CKD group vs. 1.49%, non-CKD group, *p* = 0.15), reinfarction (3.89%, CKD group vs. 1.49%, non-CKD group, *p* = 0.394), and need for new revascularization (11.68%, CKD group vs. 5.97%, non-CKD group, *p* = 0.297). As there was no difference in the endpoints between groups with long-term follow-up, this study demonstrated the safety of using intracoronary physiology to guide revascularization in patients with CKD.

## 1. Introduction

Assessment of intracoronary physiology via hyperemic or non-hyperemic methods prior to myocardial revascularization is recommended because coronary revascularization guided by intracoronary physiology reduces adverse cardiac events in patients with CAD [1]. Functional assessment can be performed using the hyperemic method (fractional flow reserve [FFR]), which requires a hyperemia-inducing drug such as adenosine, or non-hyperemic methods that do not require a hyperemia-inducing agent (instantaneous wave-free ratio [iFR], resting full-cycle ratio [RFR], and diastolic hyperemia-free ratio [DFR]). In non-hyperemic methods, the pressure gradient across the lesion is measured during the “wave-free period” of diastole, due to the lower and more stable vascular resistance in that period [2,3]. Chronic kidney disease (CKD) is a well-known risk factor for coronary artery disease (CAD) and severe CAD [4,5,6]. In patients with preexisting CAD, CKD is also associated with adverse outcomes, including increased mortality after acute coronary syndrome, myocardial revascularization with percutaneous coronary intervention (PCI), and coronary artery bypass graft surgery [7,8,9,10]. The correlation between these methods in patients with renal dysfunction and in those with preserved renal function is not well elucidated.

Our study aimed to evaluate the effectiveness of interventional strategy in patients with intermediate coronary stenosis and CKD in the Brazilian population, as a representative Latin American population.

## 2. Materials and Methods

This retrospective, single center study included consecutive patients from a region encompassing 17 municipalities from January 2018 to January 2022. This study was approved by our institutional ethics board of the Brazilian Society of Health Support Hospital. We follow STROBE reporting guidelines. Informed consent was obtained from all patients.

Consecutive patients aged > 18 years in the cited period (1) with stable angina, (2) with coronary artery stenoses with 50–80% of the vessel diameter in at least one epicardial coronary artery, and (3) with clinical data or angiographic appearance indicating or suggesting myocardial revascularization were included in this study.

Pregnant patients or those with hemodynamic instability at the time of intervention (systolic blood pressure < 90 mmHg), coronary lesions with flow < 2 after thrombolysis in myocardial infarction, contraindications for percutaneous coronary intervention or drug-eluting stent implantation, concomitant severe heart valve disease, or malignant disease with poor prognosis and a life expectancy of <1 year were excluded.

Patients found positive for ischemia using the invasive physiology method were referred for myocardial revascularization by open surgery (coronary artery bypass grafting) or coronary angioplasty (PCI), based on clinical decisions of the heart team. Drug-eluting stents were used in PCI, and patients received dual-antiplatelet therapy with aspirin and a P2Y12 inhibitor for at least six months.

For noninvasive iFR and RFR, a guidewire with a coronary pressure sensor was advanced, and the pressure was equalized with the pressure sensor at the tip of guide catheter. Subsequently, the guidewire with the pressure sensor was advanced in the coronary artery up to a point, distal to the lesion under investigation. iFR and RFR were considered positive when ≤0.89. A value between 0.86 and 0.93, based on previous studies, indicated uncertainty, and a hybrid approach with FFR was recommended depending on the operator’s preference [2,3,11]. All the patients underwent FFR with a non-hyperemic method.

We induced maximal coronary hyperemia in patients undergoing FFR with a peripheral venous infusion of adenosine (140 μg/kg per minute), and FFR was measured. PCI for stenosis was performed if the FFR was less than the ischemic threshold (0.80).

**Figure 1:**
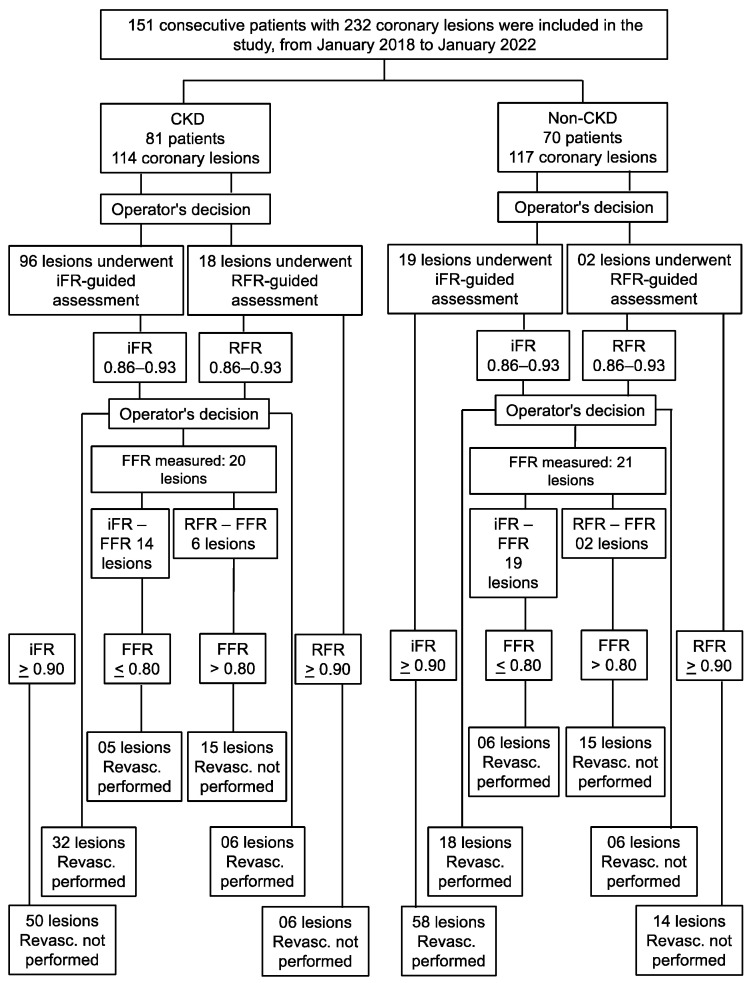
Enrollment and lesion treatment strategies. Invasive physiology was calculated for the lesions in coronary arteries with 40–80% stenosis. If the iFR/RFR value was <0.89 alone or between 0.86–0.93, followed by FFR < 0.80, then the patient was referred for revascularization and remained under optimal clinical treatment (referred lesions). If the iFR/RFR value was outside the described range, then the patient remained under optimal clinical treatment and was not subjected to any revascularization strategy (deferred lesions). FFR, fractional flow reserve; iFR, instantaneous wave-free ratio; RFR, resting full-cycle ratio.

The operators followed the flowchart shown in Figure 1. Patients were divided into 2 groups based on kidney function: patients with CKD (CKD group) and those without CKD (non-CKD group).

The iFR was calculated by measuring the pressure gradient at rest through the coronary lesion during diastole with low and stable microvascular resistance, by placing a Verrata guidewire (Philips Volcano; Rancho Cordova, CA, USA) with a pressure transducer at the distal end of the coronary lesion. 

The stages of RFR were identical to the iFR. However, we used a different guidewire, PressureWire^TM^ X guidewire (Abbott Vascular, Santa Clara, CA, USA).

The FFR was measured using the Verrata coronary guidewire (Philips Volcano, Rancho Cordova, CA, USA) or PressureWire^TM^ X guidewire (Abbott Vascular, Santa Clara, CA, USA), which was already positioned distal to the coronary artery lesion during measurement of the iFR or RFR. Adenosine was then administered to assess the pressure gradient across the lesion during maximal hyperemia. The FFR value is the ratio of pressure through the lesion during maximum hyperemia to the pressure in the aorta.

Patients who were measured by all 3 methods received intracoronary nitroglycerin and full heparinization before the guidewire was passed through the coronary artery.

The primary objective was to determine the rate of major adverse cardiac events (MACEs) during the follow-up period, which was defined as the sum of the rates of death, infarction, and need for new revascularization. The secondary endpoints were the death rate, infarction, and need for new revascularization.

Patients were followed up by face-to-face consultation or via telephonic contact at 1, 3, 6, and 12 months after the procedure and periodically thereafter. To ensure minimal misclassification of results during telephone follow-up, we implement strict quality control. The following measures were implemented. (1) We provided adequate training for the professionals involved in correctly classifying the results, including providing detailed information on the classification criteria and clear guidance on how to deal with different situations. (2) We established standardized protocols for classifying results so that all professionals were able to follow the same guidelines, thus helping to minimize variability in classifications and reduce errors arising from individual interpretations. (3) We implemented a peer review system, wherein the results for which the initial contact professionals were unsure were reviewed by another professional before being considered final; this allowed the identification of possible errors or discrepancies and guaranteed a second opinion regarding the classification. (4) To assess the accuracy of outcome classifications regular audits were performed by professionals with the use of a reference standard by randomly selecting a sample of cases and comparing the classifications. (5) We employed feedback and continuous training of professionals based on audits and review of cases in which classification errors occurred. (6) We monitored and analyzed data to identify trends or patterns of classification errors, thus helping to identify specific problem areas that require additional attention and the implementation of corrective measures. (7) We obtained feedback from patients through satisfaction surveys or interviews, allowing them to express their opinions and identify possible errors or problems.

We used the Kidney Disease Outcomes Quality Initiative and Kidney Disease: Improving Global Outcomes guidelines to define CKD as follows: a decrease in kidney function, regardless of the cause, for 3 or more months (decreased renal function as manifested by decreased glomerular filtration rate, estimated in our study using serum creatinine and Cockcroft-Gault equation [estimated glomerular filtration rate, eGFR] and expressed in mL/min/1.73 m^2^) [12].

Death was defined as death from any cause. Myocardial infarction was defined a priori in the study protocol as appearance of new pathological Q waves on electrocardiography in at least 2 contiguous leads and/or an increase in high-sensitivity troponin level > 5 times the upper limit of normality during the index hospitalization. If troponin was not measured or was not available, an increase in creatine kinase-myocardial band level > 5 times the upper limit of normality, qualified as myocardial infarction.

The need for urgent revascularization was defined as revascularization that was not part of the index procedure and not identified during the index procedure within 60 days after the procedure.

Continuous quantitative variables were assessed using a parametric Student’s *t*-test. Numerical values are presented as mean ± standard deviation. Categorical variables were compared using chi-squared test or Fisher’s exact test, as appropriate. A logistic regression model was used to adjust the variables and assess differences between groups at the time of events and outcomes. In-hospital variables at the time of index procedures were adjusted for age, sex, average body mass index, diabetes, chronic renal failure, initial clinical presentation (stable/unstable coronary artery disease), acute coronary syndrome without ST segment elevation, the coronary artery disease extent, left ventricle function, diameter stenosis, number of ischemic lesions per patient, fluoroscopy time, and volume of ionic contrast medium.

Variables at the end of the follow-up period were adjusted for follow-up loss, new revascularization, the target vessel revascularization rate, the target lesion revascularization rate (TLR), new myocardial infarction, death, and MACEs (primary outcome). Kaplan–Meier curves and log-rank tests were used to visualize the outcomes during the follow-up period, comparing the two groups.

Statistical analyses were performed using R software, version 3.3.1 (R Foundation for Statistical Computing, Vienna, Austria; https://www.R-project.org/ URL accessed on 24 February 2022). Statistical significance was set at *p* < 0.05.

## 3. Results

We recruited 151 consecutive patients from January 2018 to January 2022 from a center in the state of Bahia, Brazil and evaluated 232 lesions using invasive physiological methods. Patients were divided into 2 groups: CKD group comprising 81 patients (114 lesions) with CKD and non-CKD group comprising 70 patients (117 lesions) without CKD.

The demographic characteristics that differed between the 2 groups were as follows (Table 1): in the CKD group, mean age was higher (70.80 ± 11.41 vs. 57.47 ± 10.19 years, *p* < 0.001) and body mass index was lower (25.53 ± 4.29 vs. 27.41 ± 4.25 kg/m^2^, *p* = 0.007) than in the non-CKD group.

With regard to initial clinical presentation, there were no significant differences between the groups. About the technical characteristics, the differences were that in the CKD group, the volume of contrast used was lower (91.29 ± 84.85 vs. 123.52 ± 90.02 mL, *p* = 0.02) and fluoroscopy time was longer (81.59 ± 82.90 in the CKD group vs. 50.22 ± 57.78 min, *p* = 0.007) than in the non-CKD group. The technical characteristics of the patients are listed in Appendix A in Table A1.

The procedural characteristics related to the variables based on the number of lesions are listed in Table 2. The number of single-vessel lesions (28.39% vs. 50%, *p* = 0.07) and mild left ventricular dysfunction (6.17% vs. 18.57%, *p* = 0.03) were higher in the non-CKD group than in the CKD group. The mean iFR value tended to be lower in the CKD group than in the non-CKD group (mean value 0.90 ± 0.09 vs. 0.92 ± 0.07, *p* = 0.06). The concordance rate between non-hyperemic methods and FFR did not show a statistically significant difference between the groups. No significant differences were observed in the number of lesions or patients. The number of ischemic lesions/patient was higher in the CKD group than in the non-CKD group (0.74 ± 0.91 vs. 0.44 ± 0.70, *p* = 0.005).

The presence of CKD was strongly associated with older age (*p* < 0.001) and a higher number of ischemic lesions per patient (*p* = 0.02), after adjustment for variables using a multivariate logistic regression model. 

There was no significant difference in the mean long-term follow-up time between the groups, Appendix A, Table A2. The follow-up loss rate was 9.57% for the total sample. During the follow-up period, the primary outcomes in the CKD and non-CKD groups were 22.07% and 14.92%, respectively (*p* = 0.363). There was an initial statistical difference in TLR (11.68% in the CKD group vs. 1.49 in the non-CKD group, *p* = 0.02); analyzing the patients who presented with TLR, in the CKD group, there were 9 patients and 13 lesions (7 lesions revascularized in the index procedure and 6 were deferred [53.84% vs. 46.16%, *p* = 0.820]). In the non-CKD group, TLR occurred in only one patient with one lesion with deferred revascularization in the index procedure. The presence of a higher TLR in the CKD group was unrelated to the use of intracoronary physiology to guide revascularization; in the CKD group, practically an equal percentage of patients who did and did not have revascularization in the index procedure presented with TLR. Importantly, this initial statistical difference was not significant after adjusting for variables in the logistic regression model. This suggests that the variables other than CKD that were included in the model (described in Section 2) could better explain the relationship between the presence of CKD and the occurrence of TLR.

**Figure 2:**
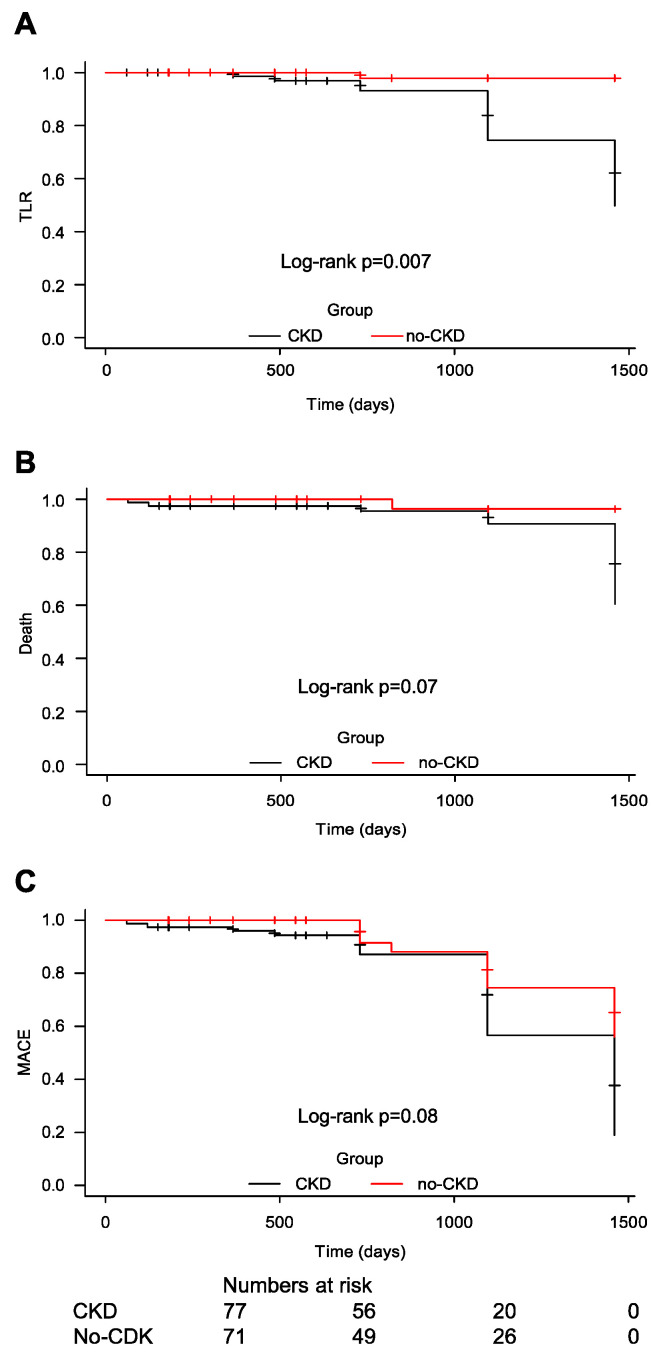
Kaplan–Meier curves for survival-free target lesion revascularization rate (**A**), major adverse cardiac events (**B**) and all-cause mortality (**C**). CKD, chronic kidney disease; MACE, major adverse cardiac event; TLR, target lesion revascularization.

Regarding the secondary outcomes, there was no difference between the rates of death from all causes (6.49% in the CKD group vs. 1.49% in the non-CKD group, *p* = 0.15), reinfarction (3.89% in the CKD group vs. 1.49% in non-CKD group, *p* = 0.394), and need for new revascularization (11.68% in the CKD group vs. 5.97% in the non-CKD group, *p* = 0.297). The Kaplan–Meier survival curves for TLR, MACEs, and death are shown in Figure 2.

## 4. Discussion

The mean iFR value tended to be lower in the CKD group; however, there was an agreement between the non-hyperemic methods and FFR. The presence of CKD was an independent predictor for older patients at the index event and a greater number of ischemic lesions per patient.

Our findings indicate that this strategy is safe and effective in patients with CKD to assess coronary physiology in a real-world setting, in a representative population from Latin America, with a mean follow-up period of 700 days. In both groups, patients had low rates of adverse cardiac events and death at late follow-up. The findings from previous studies regarding this are conflicting.

Few studies have explored the relationship between intracoronary analysis using invasive physiology and kidney disease. Previous studies have addressed issues regarding the agreement between the methods. To the best of our knowledge, our study is the second to investigate this with a long-term follow-up of safety and efficacy of this method in these patients. Our study is the only study that evaluated hyperemic and non-hyperemic methods with a long-term follow-up.

Only one previous study evaluated the long-term follow-up results using FFR in patients receiving hemodialysis after deferred revascularization, and poor results of late revascularization were observed (coronary lesions with FFR > 0.80) in these patients [13].

In our study, there was no disagreement between hyperemic and non-hyperemic methods in patients with CKD and those with normal renal function. However, we did not perform analyses between different glomerular filtration rate groups wherein previous studies have found disagreements, since only 7% of our sample of patients with CKD had eGFR < 30 mL/min/1.73 m^2^. Earlier studies have found disagreements in patients with eGFR < 30 mL/min/1.73 m^2^ and in those receiving hemodialysis.

The impact of hemodialysis on the concordance between iFR and FFR in 196 patients with 265 lesions was evaluated. A satisfactory relationship between the 2 methods was observed in patients receiving hemodialysis; however, iFR in the hemodialysis group was significantly lower than that in the non-hemodialysis group (0.81 ± 0.13 vs. 0.86 ± 0.13, *p* = 0.005). They concluded that iFR non-hyperemic method may be useful for assessment of intermediate coronary lesions in patients receiving hemodialysis. However, the threshold for iFR abnormality requires adjustment in these patients [14].

Ohashi et al. [15] classified 263 consecutive patients with 370 intermediate lesions into 3 groups according to renal function: Group 1, eGFR 60 mL/min/1.73 m^2^; Group 2, eGFR 30–60 mL/min/1.73 m^2^; and Group 3, eGFR < 30 mL/min/1.73 m^2^. The non-hyperemic method (RFR) significantly correlated with the hyperemic method (FFR) in all the groups except group 3. The authors concluded that the diagnostic performance of RFR differed based on renal function in the group with the worst renal function (eGFR < 30 mL/min/1.73 m^2^) [15].

Yamasaki et al. [16] compared the FFR and RFR in 277 patients with 408 intermediate lesions and showed that FFR and RFR were significantly correlated (r = 0.76, *p* < 0.001). Discordant results between FFR and RFR have been found in >20% of vessels with intermediate coronary lesions in patients with the following clinical features: impaired renal function and receiving hemodialysis, heart failure, and anemia [16].

Tebaldi et al. [17] reported that FFR measurements differed between patients with CKD and those with normal renal function. FFR values that indicate flow limitation were less frequent in patients with eGFR ≤ 45 mL/min; these patients had higher FFR values in this eGFR range [17].

The FIGARO Study compared FFR and iFR to assess the significance of coronary lesions and included 1564 patients and 1884 lesions. In multivariate logistic regression, they observed as predictors of disagreement: gender, age, location of the right coronary artery lesion, hemoglobin level, smoking, and renal failure [18].

Patients undergoing hemodialysis often have systemic arterial hypertension, reduced arterial compliance, left ventricular hypertrophy, and impaired microcirculation. The coronary flow in these patients may influence the relationship between hyperemic and non-hyperemic methods during evaluation of intermediate coronary stenoses and can guide clinical decisions regarding the appropriate method to use. This issue was not observed in other studies or the present study in patients with CKD and eGFR > 30 mL/min/1.73 m^2^. Thus, there is a need for better understanding of clinical factors that contribute to the discordance between these indices, including renal function, to facilitate their use in this subgroup of patients.

The main limitation of our study includes being a single-center, retrospective study and not a randomized trial. Furthermore, the number of patients was relatively modest, and some patients were lost to follow-up. Additionally, lack of absolute standardization of the operators’ conduct was a limitation, since the operators decided to perform FFR following the non-hyperemic method if its value was in the range of 0.86–0.93 or <0.89.

In conclusion, the present study showed no difference in primary (sum of infarction, new revascularization, and death) or secondary endpoints (rates of death, infarction, and need for new revascularization) between CKD and non-CKD group, demonstrating the real-world safety of this invasive physiology method as an indicator of coronary revascularization in patients with CKD.

## Figures and Tables

**Table 1:** Baseline clinical characteristics of the patients.

Variable	CKD Group (n = 81) *	Non-CKD Group (n = 70) *	*p* Value
Age (years)	70.80 ± 11.41	57.47 ± 10.19	<0.001
Female sex [n (%)]	39 (48.18)	20 (28.57)	0.101
Race			
White [n (%)]	60 (74.07)	40 (57.1)	0.32
Black [n (%)]	6 (7.4)	11 (15.71)	0.151
Brown [n (%)]	15 (18.51)	18 (25.71)	0.393
Indigenous [n (%)]	0 (0)	1 (1.42)	0.283
Average Body-mass index ^†^	25.53 ± 4.29	27.41 ± 4.25	0.007
Creatinine clearance ratio ^‡^	62.27 ± 22.55	115.98 ± 38.99	<0.001
Creatinine clearance ^‡^ ≥ 90	0 (0)	70 (46.35%)	NA
Creatinine clearance ^‡^ 60–89	41 (50.61)	0 (0)	NA
Creatinine clearance ^‡^ 30–59	35 (43.2)	0 (0)	NA
Creatinine clearance ^‡^ ≤ 29	5 (6.17)	0 (0)	NA
Hypertension [n (%)]	76 (93.82)	54 (77.14)	0.417
Dyslipidemia [n (%)]	58 (71.6)	52 (74.28)	0.883
Family history of CAD [n (%)]	41 (50.61)	29 (41.42)	0.493
Current smoker [n (%)]	7 (8.64)	10 (14.28)	0.329
Diabetes mellitus [n (%)]	30 (37.03)	25 (35.71)	0.904
Previous IM [n (%)]	27 (33.33)	26 (37.14)	0.734
Previous PCI [n (%)]	36 (44.44)	28 (40)	0.725

Data are presented as mean ± standard deviation or numbers (percentages). There were significant differences in the baseline characteristics between the two groups in age, average body mass index, and mean creatinine clearance. CKD, chronic kidney disease; CAD, coronary artery disease; IM, myocardial infarction; PCI, percutaneous coronary intervention. * Number of patients. ^†^ Body mass index is the weight in kilograms divided by the square of the height in meters. ^‡^ Creatinine clearance expressed as mL/min/1.73 m^2^ was estimated using the Cockcroft-Gault equation.

**Table 2:** Procedural characteristics related variables by the number of lesions.

Variable	CKD Group	No-CKD	*p* Value
	(n = 114) *	Group (n = 117) *	
Lesion territory [n (%)]			
Right coronary artery/branches	18 (15.78)	15 (12.82)	
Left circumflex artery/branches	19 (16.66)	19 (16.23)	
Left anterior descending artery	57 (50)	61 (52.13)	
Diagonal	8 (7.01)	13 (11.11)	0.975
Intermediate	1 (0.87)	1 (0.85)	
Saphenous vein graft	1 (0.87)	1 (0.85)	
Left internal mammary graft	1 (0.87)	1 (0.85)	
Left main	9 (7.89)	6 (5.12)	
AHA classification [n (%)]			
A	5 (4.38)	9 (7.69)	
B1	45 (4.38)	46 (39.31)	0.886
B2	23 (20.17)	22 (18.8)	
C	41 (35.96)	40 (34.18)	
Calcification [n (%)]			
Moderate	20 (17.54)	13 (11.11)	0.226
Severe	4 (3.5)	1 (0.85)	0.175
Intracoronary adenosine [n (%)]	20 (17.54)	21 (17.94)	0.946
iFR performed [n (%)] ^‡^	96 (84.21)	95 (81.19)	0.852
RFR performed [n (%)] ^‡^	18 (15.78)	22 (18.8)	0.611
FFR performed [n (%)] ^‡^	20 (17.54)	21 (17.94)	0.946
Revascularized lesions	43 (37.71)	30 (25.64)	0.154
Changed the operator’s initial decision [n (%)]	15 (13.15)	9 (7.69)	0.22
Concordant results for hyperemic and non-hyperemic methods [n (%)]			
Non-hyperemic methods followed by FFR [n (%)]	26 (22.8)	21 (17.94)	0.455
iFR followed by FFR:	20 (17.54)	19 (16.23)	0.823
iFR + followed by FFR −	1 (0.87)	5 (4.27)	0.113
iFR + followed by FFR +	2 (1.75)	3 (2.56)	0.679
iFR—followed by FFR −	15 (13.15)	9 (7.69)	0.22
iFR—followed by FFR +	2 (1.75)	2 (1.7)	0.979
RFR followed by FFR:	6 (5.26)	2 (1.7)	0.153
RFR—followed by FFR −	6 (5.26)	2 (1.7)	0.153
Diameter stenosis—mean (SD)	61.31 ± 12.44	58.11 ± 12.92	0.05
Average iFR—mean (SD)	0.90 ± 0.09	0.92 ± 0.77	0.06
Average RFR—mean (SD)	0.90 ± 0.04	0.90 ± 0.07	0.92
Average FFR—mean (SD)	0.86 ± 0.06	0.84 ± 0.04	0.41
N° of lesions assessed/patient	1.91 ± 0.87	1.82 ± 0.81	0.411
N° of ischemic lesions/patient	0.74 ± 0.91	0.44 ± 0.70	0.005

Data are presented as mean ± standard deviation or numbers (percentages). CKD: chronic kidney disease. * Number of lesions evaluated. ^‡^ Type of physiological assessment of vessels. Functionally significant lesions with an iFR/RFR ≤ 0.89 or FFR < 0.80. iFR values between 0.86 and 0.93 indicated conversion of the procedure to FFR at the operator’s discretion. AHA, American Heart Association; iFR, instantaneous wave-free ratio; FFR, fractional flow reserve. There were significant differences between the two groups in the procedural characteristics related to the number of lesions, in relation to the number of ischemic lesions per patient.

## Data Availability

All data are reported in the text.

## Appendix A

**Table A1:** Procedural characteristics related variables by the number of patients.

Variable	CKD Group (n = 81) *	Non-CKD Group (n = 70) *	*p* Value
Radial-artery approach [n (%)]	67 (82.71%)	64 (91.42%)	0.675
Volume of ionic contrast medium (mL)	91.29 ± 84.85	123.52 ± 90.02	0.02
Fluoroscopy (min)	81.59 ± 82.90	50.22 ± 57.78	0.007
Number of affected arteries ^†^			
Single vessel [n (%)]	23 (28.39%)	35 (50%)	
2-vessel [n (%)]	29 (35.8%)	20 (28.57%)	0.04
3-vessel [n (%)]	29 (35.8%)	15 (21.42%)	

Data are presented as mean ± standard deviation or numbers (percentages). CKD: chronic kidney disease. * Number of patients. ^†^ Presence of more than 50% stenosis in the epicardial coronary artery. There were significant differences between the two groups in procedural characteristics including the number of patients, volume of ionic contrast medium, fluoroscopy time, and number of affected arteries.

**Table A2:** Late follow-up.

Patients Followed [n (%)]	CKD Group (n = 81) *	Non-CKD Group (n = 70) *	*p* Value
Average segment time [mean (SD)]	727.27 ± 339.29	734.38 ± 364.66	0.903
Lost to follow-up [n (%)]	4 (5.19%)	3 (4.47%)	0.849
Repeat coronary angiography non-urgent [n (%)]	14 (18.18%)	9(13.43%)	0.508
Repeat revascularization non-urgent [n (%)]	9 (11.68%)	4 (5.97%)	0.274
TVR non-urgent [n (%)]	9 (11.68%)	3 (4.47%)	0.149
TLR non-urgent [n (%)]	9 (11.68%)	1 (1.49%)	0.02
Nonfatal IM [n (%)]	3 (3.89%)	1 (1.49%)	0.394
Death from all causes [n (%)]	5 (6.49%)	1 (1.49%)	0.15
MACE [n (%)]	17 (22.07%)	10 (14.92%)	0.363

Data are presented as mean ± standard deviation (SDs) or numbers (percentages). * Number of patients. CKD, chronic kidney disease; IM, myocardial infarction; MACE, major adverse cardiac event; TLR, target lesion revascularization; TVR, target vessel revascularization. There were significant differences between the 2 groups in terms of the rate of TLR in the late follow-up period.
